# Simultaneous Determination of 24 Antidepressant Drugs and Their Metabolites in Wastewater by Ultra-High Performance Liquid Chromatography–Tandem Mass Spectrometry

**DOI:** 10.3390/molecules19011212

**Published:** 2014-01-20

**Authors:** Ling-Hui Sheng, Hong-Rui Chen, Ying-Bin Huo, Jing Wang, Yu Zhang, Min Yang, Hong-Xun Zhang

**Affiliations:** 1 College of Resources and Environment, Graduate University of the Chinese Academy of Sciences, Beijing 100049, China; E-Mail: Shenglh@nim.ac.cn; 2 National Institute of Metrology, Beijing 100013, China; E-Mail: wj@nim.ac.cn; 3 Research Center for Eco-Environmental Sciences, Chinese Academy of Sciences, Beijing 100085, China; E-Mails: hr630@126.com (H.-R.C.); huo802@163.com (Y.-B.H.); zhangyu@rcees.ac.cn (Y.Z.)

**Keywords:** pharmaceuticals, antidepressants drug, solid phase extraction, wastewater treatment plants

## Abstract

Antidepressants are a new kind of pollutants being increasingly found in wastewater. In this study, a fast and sensitive ultra-high performance liquid chromatography-tandem mass spectrometry method was developed and validated for the analysis of 24 antidepressant drugs and six of their metabolites in wastewater. This is the first time that the antidepressant residues in wastewater of Beijing (China) were systematically reported. A solid-phase extraction process was performed with 3 M cation disk, followed by ultra-high performance liquid chromatography–tandem mass spectrometry measurements. The chromatographic separation and mass parameters were optimized in order to achieve suitable retention time and good resolution for analytes. All compounds were satisfactorily determined in one single injection within 20 min. The limit of quantification (LOQ), linearity, and extraction recovery were validated. The LOQ for analytes were ranged from 0.02 to 0.51 ng/mL. The determination coefficients were more than 0.99 within the tested concentration range (0.1–25 ng/mL), and the recovery rate for each target compound was ranged from 81.2% to 118% at 1 ng/mL. This new developed method was successfully applied to analysis the samples collected from Beijing municipal wastewater treatment plants. At least ten target antidepressants were found in all samples and the highest mean concentration of desmethylvenlafaxin was up to 415.6 ng/L.

## 1. Introduction

In recent years, more and more human-use pharmaceuticals and their metabolites have been found in waters from municipal wastewater treatment plants (WWTPs) [1,2,3,4,5]. The antidepressant drugs are a class of neuroactive compounds that act as selective serotonin reuptake inhibitors and serotonin noradrenergic reuptake inhibitors, and some of their metabolites also retain the pharmacologic activity and are capable of contributing to serotonin reuptake inhibition [6,7,8]. The unaltered drugs or their main metabolites have been detected in municipal wastewaters in many countries [9,10,11,12,13,14,15,16].

Generally, the antidepressants exist in wastewater in low concentrations. However, their potent psychoactivity cannot be neglected. There are many reports on the potential effects of such compounds that may lead to physiological and behavioral changes in aquatic organisms and accumulate in their tissues [17,18,19,20,21]. Therefore, it is important to understand the environmental profile of these antidepressants and their metabolites in order to assess their potential ecological impact [22,23]. GÒmez *et al.* analyzed the effluents from three WWTPs in Spain using solid phase extraction (SPE) with an Oasis HLB sorbent, followed by gas chromatography-tandem mass spectrometry and found that the wastewater contained 8 ng/L of carbamazepine [24]. Lajeunesse *et al.* studied the basic antidepressants and their *N*-desmethyl metabolites in raw sewage and wastewater from Montreal (Canada). Six basic antidepressants and four of their metabolites were detected by SPE and LC–MS/MS in raw sewage and roughly primary-treated wastewater with concentrations ranging from 2–346 ng/L [14]. Chen *et al.* found primidone and carbamazepine in wastewater effluent samples in Hebei Province of China with maximum concentrations of 74 ng/L and 103 ng/L, respectively [25].

To our knowledge, only a few top-selling antidepressants found in wastewater were studied in different countries [9,10,11,12,13,14,15,16]. Simultaneous analysis of the most representative antidepressant drugs such as imipramine, nortriptyline, clozapine, mirtazapine, and their metabolites has not been reported up to now. The aim of this study is to investigate the occurrence of representative antidepressant drugs in wastewater from WWTPs in Beijing, China, which may facilitate a better understanding of the potential ecological and human health risks of these antidepressants and their metabolites in wastewater.

## 2. Results and Discussion

### 2.1. Optimization of LC–MS/MS

It has been shown that mass spectrometry is the most suitable tool for determination of trace environmental pollutants due to its high selectivity and sensitivity. Because most antidepressant drugs contain nitrogen-containing moieties with high proton affinities, in this study positive electrospray ionization (+ESI) was employed to analyze these antidepressant pharmaceuticals. Full-scan and MS/MS mass spectra of each compound were obtained by a 5500 Qtrap Tandem Mass Spectrometer *via* infusion of 50 ng/L individual standard solutions at a flow rate of 10 ìL/min. Two MRM transitions (quantifier and qualifier) were selected for each compound except norfluoxetine, fluoxetine and duloxetine, for which only one transition was monitored due to the poor fragmentation. The declustering potential (DP), collision energy (CE) and collision cell exit potential (CXP) for each analyte were optimized through direct infusion into mass spectrometer at the flow rate of 10 ìL/min. The optimized parameters of each mass transition were listed in Table 1.

**Table 1:** Mass spectrometer parameters for each analyte.

Analyte	Precusor ion (m/z)	Product ion (m/z)	Declusteringpotential (V)	Collision energy (V)	Collision Exit potential(V)
Citalopram	325.2	262.1 *	110	26	17
		234		36	
Sertraline	306.2	275 *	30	16	13.8
		159		34	
Venlafaxine	278.2	58 *	60	20	9
		121		34	
Desmethyl-	264.2	58 *	65	50.7	9
venlafaxine		107		49.7	
Fluvoxamine	319.2	71 *	30	18.3	16
		200		26.9	
Desmethyl	305.1	71 *	44	15	13
Fluvoxamine		200		32	
Fluoxetine	310	44 *	40	54	18.9
Norfluoxetine	296.2	134 *	37	8.5	11.2
Paroxetine	330.2	192 *	100	28	18
		70.2		50	8
Duloxetine	298.2	154 *	40	7.4	17
Mirtazapine	266.1	195 *	60	28.5	15.6
		72		26.2	
Desmethylmirtazapine	252	195 *	60	30.9	17.9
		209		28.6	
Trazodone	372.3	148 *	80	42	14
		176		31	
Clomipramine	315.2	86 *	66	22.9	10
		58		70	
Norclomipramine	301.1	72 *	56	20	15
		242		30	
Clozapine	327.1	270 *	34	35	32.1
		296		35	
Demethyl-	313.1	270 *	100	33	15
clozapine		227		38	
Quetiapine	384.2	253 *	110	30	19
		221		50	
Olanzapine	313.1	256.1 *	80	29.7	20
		198		50	
Imipramine	281.1	86 *	66	23.5	9
		58		59.8	
Bupropion	240.1	184 *	50	16.5	14
		131		35	
Amitriptyline	278.2	91 *	80	29	13
		233.2		23	
Milnacipran	274.1	100 *	30	25	12
		230		15	
Nortriptyline	264.2	91 *	100	27	10
		105		26	
Doxepin	280.2	107.1 *	58	28	13
		141		31.5	
Nordoxepin	266.1	107 *	83.4	24.9	13
		91		27	
Fluphenazine	438.2	171.1 *	60	32.3	12.2
		143.1		39.2	
Moclobemide	269.1	182 *	60	27.4	11
		139		39.6	
Chlorpromazine	319.1	86 *	85	24	17
		58		53	
Mianserin	265.2	208 *	130	28	18
		118		35	

* Quantitative ion.

Since antidepressants and their metabolites exhibit basic characteristics due to their amine moieties, a source of protons in solution is essential to convert the basic substances into their cationic forms. In the present study, to improve ionization during the electrospray process, different concentrations of the mobile solutes ammonium formate and ammonium acetate in acetonitrile were tested. Meanwhile, the pH of the buffer was also evaluated. The highest signals sensitivity was observed when using ammonium acetate (10 mM, pH 3.0) as the mobile phase.

**Figure 1:**
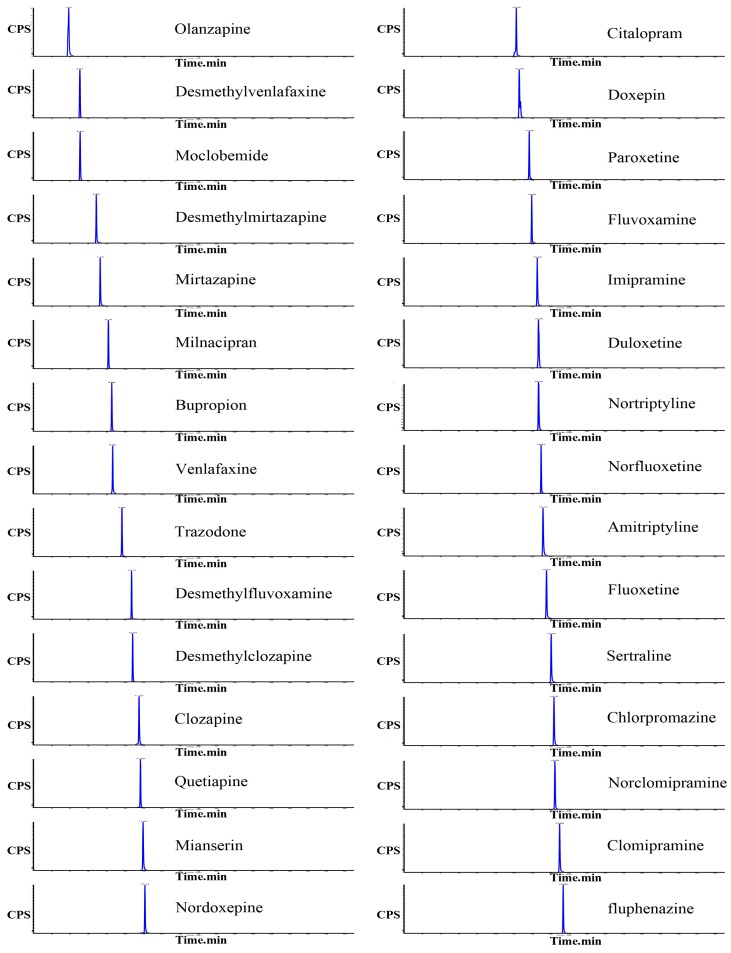
MRM chromatogram of 30 compounds obtained from a mixture of standard samples.

In order to get a good separation of all analytes, the retention and separation ability of CSH C18, HSS T3 C18 and BEH C18 columns were tested under the selected chromatographic conditions. The optimal peak shapes and resolution were achieved with BEH C18 column. The representative ion chromatograms of each compound obtained from a mixture of 30 standard samples are shown in Figure 1.

### 2.2. Solid-Phase Extraction (SPE) Study

To extract the target analytes from aqueous samples and reduce coextractive impurities from matrixes, two kinds of SPE sorbents, *i.e.*, Oasis HLB and 3 M Empore cation, were compared. The recovery percentage was used to estimate the SPE process efficiency for the selected analytes. In the case of HLB, which was used in most studies of trace drugs, the recovery was low, especially for olanzapine (44%) and clozapine (40%).

In contrast, the antidepressants and their metabolites were efficiently retained with higher recovery on 3 M cation disk. The higher recovery on 3 M cation disk was due to the filled cation-exchange sorbents which provide more effective sample clean-up for basic drugs by protonation of the free basic group. In the present study, the relative recovery rate for each target compound was from 81.2% to 118% at 1 ng/mL. Therefore, the 3M cation disk was chosen for SPE because of its superior extraction recoveries for all analytes in the wastewater.

### 2.3. Method Validation

The method was validated for linearity, limit of detection (LOD) and limit of quantification (LOQ) by the analysis of spiked milli-Q water samples. The results are listed in Table 2. In each case, a weighted linear regression line was applied. The LOD and LOQ were calculated as three and ten times signal to noise ratio at the 1 ng/mL concentration with an accuracy ranged from 80% to 120% and precision within ± 15% of the target concentration. The LODs ranged from 0.01 to 0.15 ng/mL and the LODs of norclomipramine, clozapine, quetiapine, nordoxepin and chlorpromazine were the lowest (0.01 ng/mL). The determination coefficient (*r^2^*) of all calibration curves was more than 0.99 within in the tested concentration range (0.1–25 ng/mL). Both selectivity and sensitivity of the established method were satisfactory, and no interfering substances presented at the appropriate retention times.

**Table 2:** Correlation coefficient, linearity range, LOD and LOQ of the method.

Analyte	Correlation coefficient	Concentration range (ng/mL)	LOD (ng/mL)	LOQ (ng/mL)
Citalopram	0.9988	0.1–10	0.04	0.11
Sertraline	0.9989	0.1–10	0.02	0.06
Venlafaxine	0.9987	0.1–10	0.03	0.09
Desmethylvenlafaxine	0.9979	0.1–10	0.05	0.15
Fluvoxamine	0.9992	0.1–10	0.07	0.23
Desmethylfluvoxamine	0.9998	0.5–25	0.09	0.32
Fluoxetine	0.9986	0.1–10	0.12	0.50
norfluoxetine	0.9998	0.5–25	0.15	0.51
Paroxetine	0.9990	0.1–10	0.05	0.16
Duloxetine	0.9980	0.1–10	0.04	0.12
Mirtazapine	0.9989	0.1–10	0.02	0.06
Desmethylmirtazapine	0.9989	0.1–10	0.03	0.10
Trazodone	0.9995	0.1–10	0.02	0.06
Clomipramine	0.9995	0.1–10	0.03	0.08
Norclomipramine	0.9985	0.1–10	0.01	0.04
Clozapine	0.9976	0.1–10	0.01	0.04
Demethylclozapine	0.9986	0.1–10	0.05	0.16
Quetiapine	0.9991	0.1–10	0.01	0.03
Olanzapine	0.9973	0.1–10	0.05	0.17
Imipramine	0.9996	0.1–10	0.02	0.07
Bupropion	0.9997	0.1–10	0.04	0.13
Amitriptyline	0.9971	0.1–10	0.03	0.10
Milnacipran	0.9983	0.1–10	0.07	0.21
Nortriptyline	0.9985	0.1–10	0.03	0.10
Doxepin	0.9949	0.1–10	0.04	0.13
Nordoxepin	0.9977	0.1–10	0.01	0.04
Fluphenazine	0.9975	0.1–10	0.03	0.10
Moclobemide	0.9993	0.1–10	0.05	0.15
Chlorpromazine	0.9962	0.1–10	0.01	0.03
Mianserin	0.9986	0.1–10	0.04	0.14

### 2.4. Application to Environmental Analysis

This method was applied to detect antidepressants and their metabolites in the wastewater from three WWTPs in Beijing. The compounds were detected and confirmed by comparing their retention time and MRM transitions Eight antidepressants and two metabolites were successfully found in all wastewater samples. The concentrations of the antidepressants and metabolites are listed in Table 3. The metabolite desmethylvenlafaxine was found at the highest concentration of (415.6 ± 32.9) ng/L, while the desmethylmirtazapine was detected in the effluents of WWTPs at the lowest concentration of (4.0 ± 0.2) ng/L. The measured concentrations of these antidepressants and their metabolites were consistent with the previous reports about wastewaters [14,26].

**Table 3:** Concentrations (ng/L) of antidepressants and their metabolites in wastewaters (n = 3).

Analyte	WWTP1#	WWTP2#	WWTP3#
Citalopram	20.6 ± 2.0	18.3 ± 2.0	28.8 ± 3.5
Sertraline	37.3 ± 1.3	40.6 ± 4.0	18.8 ± 2.8
Venlafaxine	31.8 ± 4.3	63.7 ± 2.6	30.3 ± 4.6
Desmethylvenlafaxine	52.3 ± 2.7	71.3 ± 4.8	415.6 ± 32.9 ^a^
Mirtazapine	62.8 ± 4.8	76.3 ± 5.8	84.2 ± 13.7
Desmethylmirtazapine	4.0 ± 0.2	7.3 ± 1.3	10.1 ± 1.5
Clomipramine	101.7 ± 11.6 ^a^	77.5 ± 4.3	92.3 ± 10.7
Clozapine	91.7 ± 4.3	53.4 ± 4.8	163.9 ± 17.7 ^a^
Imipramine	10.8 ± 0.6	10.6 ± 2.1	10.9 ± 1.1
Nortriptyline	47.8 ± 1.3	35.1 ± 5.1	43.8 ± 7.8

a: The concentration was calculated from a diluted (1:10) sample.

## 3. Experimental

### 3.1. Chemicals and Reagents

All solvents were of HPLC grade obtained from Merck (Darmstadt, Germany). Ammonium acetate and ammonium formate were purchased from Sigma–Aldrich (St. Louis, MO, USA). Chemical standards of the antidepressant drugs (citalopram, sertraline, venlafaxine, fluvoxamine, fluoxetine, paroxetine, duloxetine, mirtazapine, trazodone, clomipramine, clozapine, quetiapine, olanzapine, imipramine, bupropion, amitriptyline, doxepin, fluphenazine, moclobemide, mianserin, milnacipran) were purchased from National Institutes for Food and Drug Control of China (Beijing, China). Desmethyl -venlafaxine was purchased from Zibo Dingjin Chemical Co., Ltd (Shandong, China). Norfluoxetine, desmethylmirtazapine, desmethylvenlafaxine, desmethylfluvoxamine, chlorpromazine, demethylclozapine, norclomipramine, nortriptyline were purchased from Toronto Research Chemicals Inc. (North York, ON, Canada). Standard stock solutions of these compounds were prepared in methanol (1 mg/mL). Deionized water was purified using a Milli-Q water purification system (Merck Millipore, Bedford, MA, USA).

Water samples were collected between December 2012 and April 2013 from wastewater treat plants of Beijing. The water samples were vacuum filtered through 1μm glass fiber filters, followed by 0.22 μm nylon membrane filters right after the sampling, and stored on −40 °C until the analysis.

### 3.2. Apparatus and Operation Conditions

The chromatographic separations were performed using the Agilent 1290 UHPLC system (Agilent, Waldbronn, Germany) equipped with a BEH C18 column, 2.1 × 150 mm, 1.7 μm (Waters Corporation, Milford, MA, USA). Gradient elution was applied using 10 mM ammonium acetate containing 0.1% formic acid (A) and acetonitrile (B) as the mobile phase and programmed as follows: the gradient started with 20% eluent B in 5 min and increased linearly up to 60% in 15 min. Then, it decreased linearly again down to 20% in 5 min. The flow rate was 0.3 mL/min.

All the samples were analyzed with a 5500Qtrap Tandem Mass Spectrometer (Applied Bioscience, Foster City, CA, USA). Quantification was achieved by using multiple reaction monitoring (MRM). The declustering potential (DP), collision energy (CE) and collision cell exit potential (CXP) for each compound were optimized, which are listed in Table 1. Additional instrumental parameters for all analytes were as follows: curtain gas (CUR) setting at 45 psi, spray voltage at 4,500 V, source temperature at 600 °C, gas1 at 60 psi and gas2 at 50 psi.

### 3.3. Sample Extraction

To remove suspended material, the water samples were vacuum filtered through 1 μm glass fiber filters, followed by 0.22 μm nylon membrane filters right after the sampling. The pH of each 300 mL of filtered sewage was adjusted to around 3 with hydrochloric acid (6 mol/L). An automated solid-phase extraction system Dex4790 (Horizon Technology, Salem, NH, USA) and 47 mm cation disks (3M Corporation, St. Paul, MN, USA) were employed. SPE was performed at flow rates 30–40 mL/min. Before the sample loading, the disk was washed with 8 mL of methanol, 8 mL of Milli-Q water, and 8 mL of acidified (pH 3) water. Samples were loaded onto the disk by gravity, and then the disk was washed with 10 mL of Milli-Q water, 10 mL 10% methanol and vacuum dried for 5 min. Finally the target drugs were eluted using 12 mL of 8% ammonia solution in methanol. The eluate was evaporated to dryness at 40 °C under a gentle stream of nitrogen and reconstituted in 1 mL of 20% acetronitrile solution.

## 4. Conclusions

A method based on the application of UHPLC–MS/MS for the simultaneous determination of 24 antidepressants and six metabolites in WWTP wastewaters was developed and validated. This is the first time the determination of up to 30 psychoactive compounds in one injection is reported, and 10 compounds were determined in all the effluents from three WWTPs in Beijing. Of these analytes, four antidepressants and two metabolites (venlafaxine, desmethylvenlafaxine, mirtazapine, desmethylmirtazapine, imipramine, nortriptylin) were found for the first time. High sensitivity and lower LOD were obtained thanks to the use of characteristic transitions. This study revealed the prevalence of antidepressants and their metabolites in wastewater effluents. The method developed in this study proved to be a valuable tool in the analytical characterization of antidepressants and their metabolites in wastewater, and may be helpful for determination of these drugs in sediment samples.
